# In Vitro and In Planta Studies on Temperature Adaptation of *Exserohilum turcicum* Isolates from Maize in Europe and South America

**DOI:** 10.3390/pathogens10020154

**Published:** 2021-02-02

**Authors:** Barbara Ludwig Navarro, Raphael de Araújo Campos, Maria Cândida de Godoy Gasparoto, Andreas von Tiedemann

**Affiliations:** 1 Department of Crop Sciences, Division of Plant Pathology and Crop Protection, Georg-August-Universität Göttingen, Grisebachstraße 6, 37077 Göttingen, Germany; atiedem@gwdg.de; 2 UNESP-São Paulo State University, Campus of Registro, Av. Nelson Brihi Badur, 430, Registro 11900-000, Sao Paulo, Brazil; campos.ra@hotmail.com (R.d.A.C.); maria.candida@unesp.br (M.C.d.G.G.)

**Keywords:** *Setosphaeria turcica*, aggressiveness, leaf disease, climate warming

## Abstract

Northern Corn Leaf Blight (NCLB) is a fungal leaf disease in maize caused by *Exserohilum turcicum*. NCLB occurs worldwide, from tropical to temperate zones raising the question about plasticity of temperature adaptation of local isolates of the pathogen. Seven isolates of *E. turcicum* originating from South America and seven from Europe were compared for their response to temperature variations in vitro and in vivo between 15 and 30 °C. In vitro, isolates originating from Europe and South America significantly differed in mycelial growth rate at 30 °C and in sporulation at 25 °C and 30 °C. Aggressiveness of *E. turcicum* isolates was evaluated on three susceptible maize cultivars (maize lines B37, Sus1 and the German hybrid Niklas) under different day/night temperature regimes (15/10 °C, 20/15 °C, 25/20 °C, or 30/25 °C) with a photoperiod of 14 h. Aggressiveness, recorded as area under the disease progress curve (AUDPC), of South American isolates was higher than for European isolates at 15 °C, 20 °C and 25 °C, and for sporulation in vivo in all temperatures. In general, aggressiveness components were most influenced by temperature. Therefore, multivariate analysis was performed with aggressiveness component data at 30 °C, which expressed the highest number of variables with significant differences between isolate origins. According to their aggressiveness, European and South American isolates can be grouped separately, demonstrating that South American isolates are better adapted to higher temperatures and display a higher level of aggressiveness under similar conditions than European isolates from a cool climate. It is concluded that plasticity of temperature adaptation in *E. turcicum* populations is relatively large and allowed *E. turcicum* to follow the recent expansion of maize cultivation into cool climate zones in Europe. However, our data suggest that adaptation to higher temperature is likely to increase aggressiveness of NCLB on maize in cooler climate zones when experiencing further climate warming. This plasticity in adaptation to environmental conditions of *E. turcicum* may also hamper the success of breeding programs as it may decrease the durability of resistance.

## 1. Introduction

*Exserohilum turcicum*, the causal agent of Northern Corn Leaf Blight (NCLB), occurs in all maize-producing regions, from tropical to temperate zones [1]. The ascomycete *E. turcicum* (teleomorph *Setosphaeria turcica*) causes cigar-shaped green-grey lesions on leaves, which become necrotic in later infection stages and may evolve to blight symptoms, leading to high yield losses in maize. Yield losses caused by NCLB are correlated with host phenological stage, insertion of the infected leaves and host resistance. Infections that occur from 2 to 3 weeks after pollination cause yield losses of up to 40% [2]. In the vegetative stage, young seedlings usually present higher NCLB susceptibility when compared to 2-month-old plants [3]. Lesions on the leaf closest to the cob show a high contribution to yield reduction [4].

Maize yield losses caused by *E. turcicum* are up to 40% in South America [5,6]. In Germany, *E. turcicum* causes yield losses from 10 to 30% in maize production, depending on the host resistance levels [7]. Maize-producing regions in South America are classified as Cfa (temperate climate without dry season and with hot summer), Cfb (temperate climate without dry season, with warm summer) or Cwa (temperate climate with dry winter and with hot summer) according to the Köppen-Geiger climate classification [8]. In Europe, maize-producing regions have a mild climate, Cfb, or Dfb (cold climate without dry season, with warm summer) [8]. Furthermore, control methods and cropping systems differ between the regions. Disease control in South America is mainly based on fungicide sprays and resistant cultivars. In Brazil, 28 fungicides are registered for NCLB control [9]. Nonetheless, the cultivation of susceptible genotypes in areas with weather conditions favorable for disease development and the use of no-till practices have increased disease pressure in some regions of South America [6]. In Germany, the cultivation of resistant cultivars is recommended for NCLB control. In this country, fungicide sprays are unusual, and only one fungicide was registered for NCLB control in 2020 [7].

The range of *E. turcicum* races in Europe seems to be different from that in South America. In 2011 and 2012, 10 races were reported in Europe: 0, 1, 3, 3N, 13, 23, 123, 2, 12, 13N, and 1N [10]. In contrast, race 0 is predominant in South America [11]. However, isolates capable of overcoming the resistance conferred by *Ht* gene (races 1N, 12N, 123N, 123, 23) were also detected in maize plants during 1993, 1994 and 2005 in Brazil [12,13].

Environmental conditions favorable for the occurrence of NCLB in the field are long dew periods and moderate temperatures [14]. Conidia can germinate from 10 to 35 °C and reach 100% germination from 20 to 25 °C after 2 h of dew [3]. As *E. turcicum* penetrates directly through the epidermis [15], the optimal temperature is also required for appressorium formation. Infections occur from 15 to 30 °C, and the optimal temperature is 20 °C. A minimum dew period of 5 h is required for lesion formation. In addition, the minimum dew period required for spore production is 9 h, which is longer than that necessary for infection [3,16]. Optimal weather conditions described for disease development are observed in mid-altitude regions in the tropics [14]. Accordingly, it is hypothesized that the *E. turcicum* center of origin is in the tropical regions, which is also supported by the higher genetic diversity found in those areas [17]. If the pathogen co-evolved with maize (*Zea mays*), the center of origin should be Central America. If it co-evolved with sorghum (*Sorghum bicolor*) and later jumped to maize, the center of origin should be East Africa [18].

Aggressiveness designates the amount of disease caused by one pathogen isolate on a susceptible host [19]. The aggressiveness level is related to the pathogen, but also to the host quantitative resistance and the environmental conditions [20]. Resistant hosts tend to select more aggressive isolates than susceptible hosts [21]. The interaction between host resistance and pathogen genotype plays an important role in the durability of quantitative resistance [22]. Pathogen populations with a fast response to selection pressure may erode resistance more rapidly [21].

Aggressiveness is quantified by the evaluation of components related to the disease cycle [23], such as incubation period, disease severity, and sporulation [24]. These disease components allow quantitative comparisons between pathogen isolates from tropical and temperate climate zones. In tropical regions, high temperatures and short dew periods are unfavorable to conidium survival and germination. As weather conditions in tropical regions are not always favorable for disease spread, tropical pathogens usually have an alternative source of propagation by lesion expansion [25]. Therefore, pathogen development continues by leaf tissue colonization (autoinfection) instead of conidial propagation [25]. Such a strategy is observed for *E. turcicum*, as lesion expansion has been proven to contribute in NCLB epidemics [26,27]. However, comparisons between pathogen populations originating from areas with different environmental conditions are scarce in the literature [28].

Assuming co-evolution with either maize or sorghum [18], *E. turcicum* should be adapted to tropical temperature levels. However, maize production has expanded substantially into cooler climate zones like Germany in the last three decades, exposing potential pathogens to cooler temperature regimes. This raises the question as to whether and how adaptation to cooler temperatures has affected the aggressiveness of *E. turcicum* on maize. A study of aggressiveness with *E. turcicum* isolates from Europe and Africa carried out on detached maize leaves, however, did not confirm different aggressiveness levels according to the isolate origin [29]. In the present study, a comprehensive comparison of the aggressiveness of *E. turcicum* isolates under different temperature conditions was performed. Experiments were carried out to verify the effect of temperature on pathogen and disease development, to better understand temperature adaptation of *E. turcicum* isolates originating from tropical and temperate climate zones. The effect of temperature was evaluated on mycelium growth and spore production in vitro and on incubation period, disease severity, and pathogen sporulation on maize plants in vivo.

## 2. Results

### 2.1. Effect of Temperature and Isolate Origin on Pathogen Development In Vitro

**Figure 1:**
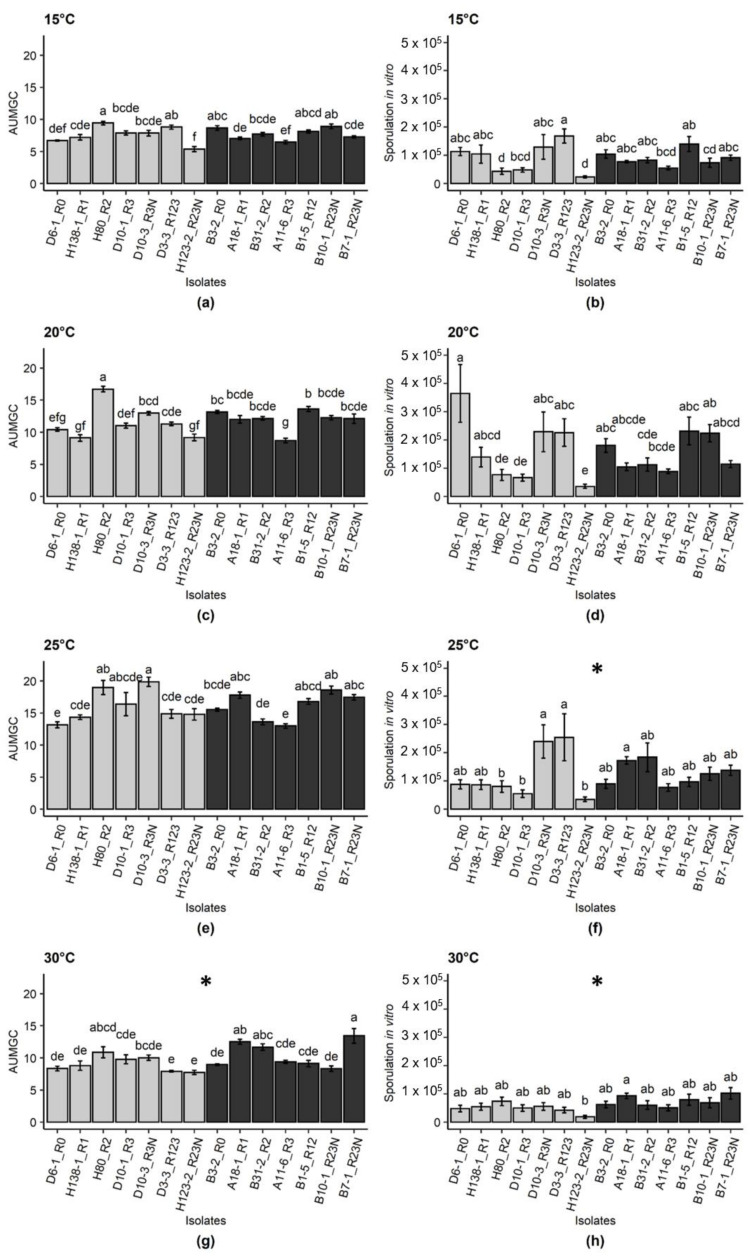
Area under the mycelium growth curve (AUMGC) (**a**,**c**,**e**,**g**) and spore production in vitro (conidia production per plate at 15 days post inoculation–dpi) (**b**,**d**,**f**,**h**) for isolates of *Exserohilum turcicum* originating from Europe and South America. Light grey bars represent European isolates, dark bars represent South American isolates. Means sharing the same letter are not significantly different following a Tukey test (*p*-value ≤ 0.05). Graphs labelled with an asterisk (*) indicate that values for the respective variable were significantly higher for South American isolates than European isolates for the analysis of variance (ANOVA, *p*-value ≤ 0.05). Bars indicate standard errors. Data are pooled from four replicated plates for each isolate which was repeated four times (*n* = 16 plates per isolate).

Independent of the isolate origin, the optimal temperature for the pathogen growth was 25 °C, followed by the temperatures 20 °C, 30 °C and 15 °C in this order, according to the area under the mycelium growth curve (AUMGC) data (Figure 1). Mycelium growth statistically (*p*-value ≤ 0.05) differed between isolates originating from Europe and South America at 30 °C. At 15 days post inoculation, the optimal temperature for spore production in vitro was 20 °C, with a mean of 156,631 conidia per plate, while at 25 °C and 30 °C, the average conidia production was 123,292 and 61,248 conidia per plate. South American isolates sporulated at a higher rate than European isolates at 25 °C and 30 °C (*p*-value ≤ 0.05) (Figure 1). Considering all temperatures, the average sporulation for South American isolates was 110,064 conidia per plate and for European isolates 105,485 conidia per plate. At 25 °C and 30 °C, the mean sporulation for South American isolates was 126,433 and 73,586 conidia per plate, whereas for European isolates mean values of 120,151 and 48,909 conidia per plate were observed, respectively.

### 2.2. Effect of Isolate Origin, Temperature and Host Genotype on Incubation Period, Disease Severity and Sporulation

**Figure 2:**
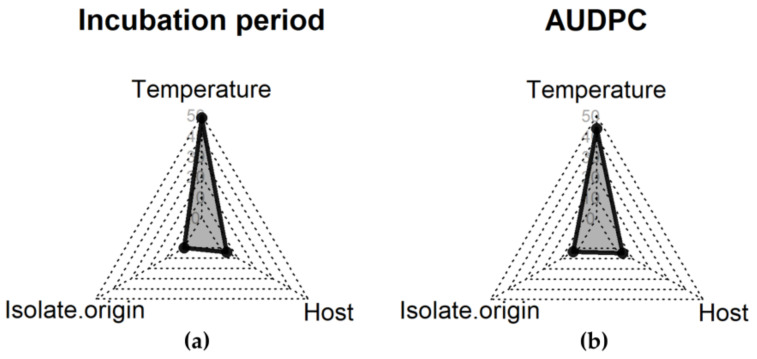
Effect of temperature, isolate origin and host genotype on total variance of incubation period (**a**), and area under the disease progress curve (AUDPC) (**b**), evaluated in the in vivo experiments in the greenhouse with three maize cultivars. The axis indicates the percentage share of total variance. Variance component analysis (VCA) was estimated by the restricted maximum likelihood method.

The effects of temperature, isolate origin, and host were explained for incubation period, and area under the disease progress curve (AUDPC), evaluated in the in vivo experiments. These variables showed the highest variance for the factor temperature. The temperature explained 48.6% and 43.7% of the total variance for incubation period, and AUDPC, respectively. All other factors explained less than 5% of the total variance (Figure 2). Therefore, isolates were compared between their origins for each temperature.

**Figure 3:**
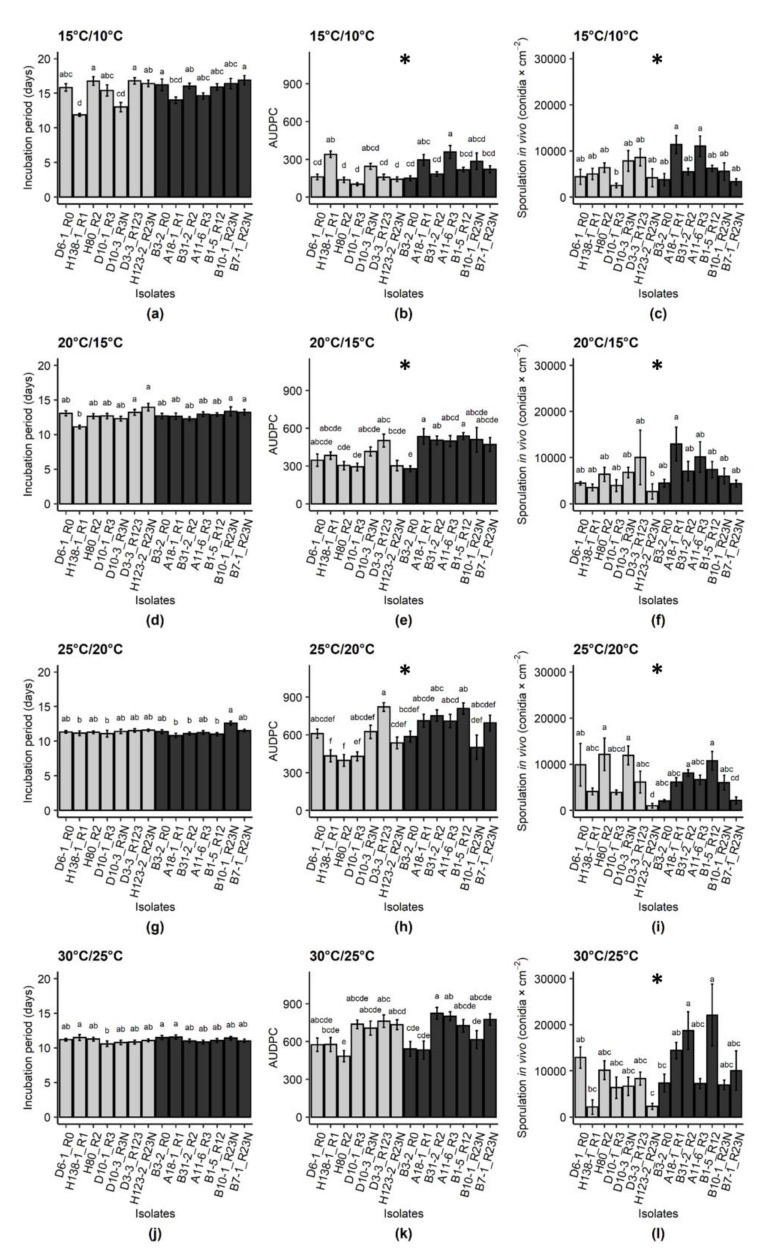
Incubation period (**a**,**d**,**g**,**j**), area under the disease progress curve (**b**,**e**,**h**,**k**) and conidia production in vivo [conidia×cm^−2^ of diseased leaf area] (**c**,**f**,**i**,**l**) for isolates of *Exserohilum turcicum* originating from Europe and South America. Light grey bars represent European isolates, dark bars represent South American isolates. Means sharing the same letter are not significantly different following Tukey test (*p*-value ≤ 0.05). Graphs labelled with an asterisk (*) indicate that values of the respective variable for South American isolates were significantly higher than for European isolates (*p*-value ≤ 0.05). Bars indicate standard errors (*n* = 24 plants). Each experiment was replicated two times.

The incubation period was longer at the coldest temperature 15/10 °C, with a mean of 15.4 days (Figure 3). At 20/15 °C, 25/20 °C and 30/25 °C, the average incubation periods were 12.7, 11.3, and 11.1 days, respectively. There was no significant difference in incubation periods between isolates originating from Europe and South America (analysis of variance (ANOVA, *p*-value = 0.072). (Figure 3), showing mean values of 12.5 and 12.7 days, respectively. The AUDPC of South American isolates was significantly higher at 15/10 °C, 20/15 °C and 25/20 °C (Figure 3). In general, South American isolates displayed higher mean AUDPC, when compared to European isolates. The sporulation in vivo of South American isolates was higher than of European isolates at all tested temperatures (Figure 3), with means of 8768.09 conidia×cm^−2^ vs. 5898.67 conidia×cm^−2^, respectively.

**Figure 4:**
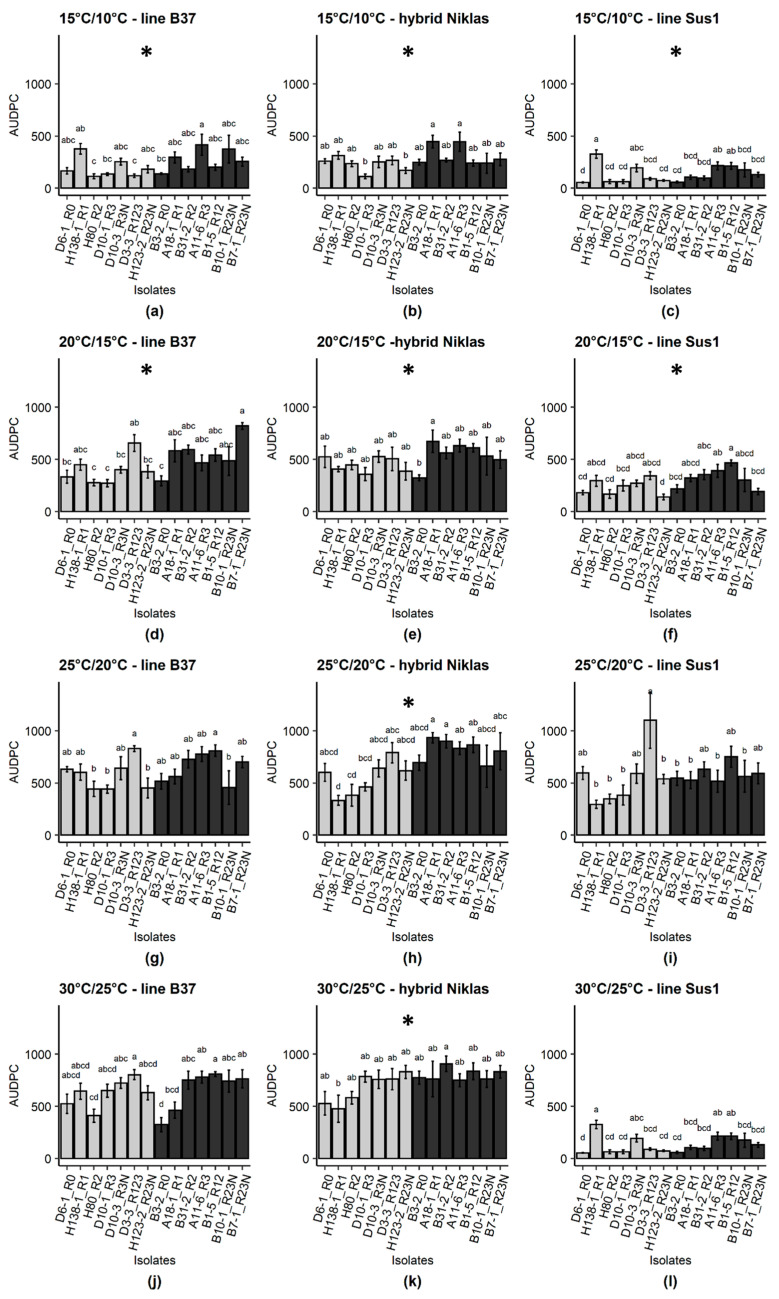
Area under the disease progress curve (AUDPC) of *Exserohilum turcicum* isolates originating from Europe and South America. Effect of host genotypesB37–standard line for race monitoring (**a**,**d**,**g**,**j**), Niklas^®^–susceptible hybrid cultivated in Germany (**b**,**e**,**h**,**k**), and Sus1–susceptible breeding line (**c**,**f**,**i**,**l**). Light grey bars represent European isolates, dark bars represent South American isolates. Means sharing the same letter are not significantly different following Tukey test (*p*-value ≤ 0.05). Graphs labelled with an asterisk (*) indicate that values for the respective variable of South American isolates were significantly higher than for European isolates (*p*-value ≤ 0.05). Bars indicate standard errors (*n* = 8 plants). Each experiment was replicated two times.

For the interaction of isolate origin × host genotype, differences in AUDPC between European and South American isolates were observed on the maize line B37 (*p*-value ≤ 0.01) and the German hybrid line Niklas (*p*-value ≤ 0.001). With regard to the temperature, higher AUDPC values were recorded for South American isolates at 15/10 °C, and at 20/15 °C for the maize lines B37 and Sus1 (Figure 4, Appendix A-Figure A1). On the maize hybrid line Niklas, South American isolates caused high AUPDC values under all tested temperatures.

### 2.3. Relation between Aggressiveness Components and Isolate Groups

**Figure 5:**
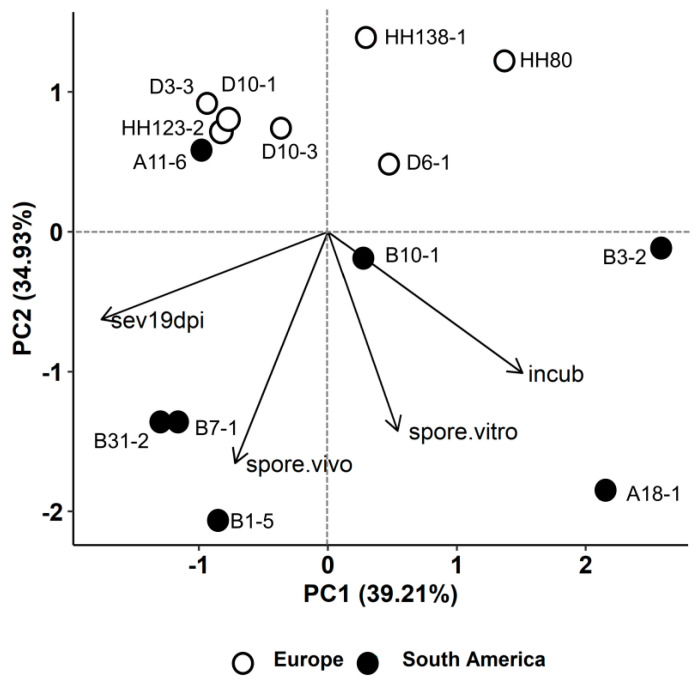
Principal component analysis (PCA) of the variables selected in the discriminant analysis (DA): disease severity at 19 days post inoculation–dpi (sev19dpi), sporulation in vivo (spore.vivo), spore production in vitro (spore.vitro), and incubation period (incub). Fourteen isolates of *Exserohilum turcicum* originating from Europe and South America were included in the analysis. Variable loadings and isolates scores are represented for the reference line B37 at 30 °C.

Further analysis of effects of isolate origin were performed with data of the AUMGC, sporulation in vitro, incubation period, AUDPC, sporulation in vivo, disease severity at 19 dpi and disease severity at 26 dpi from the reference line B37 maintained at 30/25 °C (30 °C for the experiment in vitro) to exclude the effect of host genotype and temperature. A discriminant analysis (DA) was performed to select the variables that were most contributing to distinguish isolates according to their origin. DA retained the variables incubation period, disease severity at 19 dpi, sporulation in vivo, and spore production in vitro for isolate discrimination according to their origin. However, even performing DA with the selected variables, the DA misclassified the South American isolate A11-6, which was also observed in the principal component analysis (PCA) conducted with these four DA selected variables (Figure 5). According to the PCA analysis, the components PC1 and PC2 explained 74.14% of the total variability. Except for the South American isolate A11-6, a clear distinction between South American and European isolates was observed.

## 3. Discussion

The underlying concept of this study follows the ‘disease triangle’ [30,31] considering the factors of temperature, host genotype and isolate aggressiveness putatively determined by the origin. Under controlled conditions, fungal vigor and disease components were most influenced by temperature, which was responsible for the highest variance. In most pathosystems, an increase in temperature is positively associated with aggressiveness [32,33]. As environmental conditions and host genotype usually strongly correlate with disease [34], the host genotype factor with three levels (reference line B37, susceptible line Sus1, and the hybrid Niklas^®^) was included in the experimental design. Interestingly, an interaction between isolate origin and host genotype was observed for the variable AUDPC. Disease severity was higher when South American isolates were inoculated in the hybrid Niklas than European isolates (Figure 4, Appendix A-Figure A1). It is probable that European hybrids have been selected by breeding programs according to their responses to European pathogen populations. However, all isolates were virulent in all tested host genotypes, indicating that the tested lines and hybrid do not harbor any known *Ht* genes. Usually, a pathogen population is more aggressive to a host population from the same region [24], or even more aggressive in cultivars that they were isolated from in the field [35], indicating adaptive shifts. Thus, aggressiveness can be correlated with genetic background and may have some specificity to the host genotype [22,24].

Data for mycelium growth represent the effect of temperature on pathogen vigor. The interaction between temperature and isolate origin was significant for the variable AUMGC (Figure 1). South American isolates showed higher mycelium growth at 30 °C indicating that they might be more adapted to higher temperatures than European isolates. A study carried out with *Sclerotinia sclerotiorum* isolates showed a similar result. Isolates collected from warmer areas were better adapted to higher temperatures, and isolates from colder areas were more adapted to colder temperatures [28]. In general, plant pathogens adapt to changes in environmental conditions by phenotypic plasticity, migration to areas with more favorable climatic conditions or mutations in their genomes, which all favor pathogen survival. Plasticity consists of the ability to adapt without the need for mutation. Plasticity might be correlated to a population with higher genetic diversity, as tropical *E. turcicum* populations are genetically more diverse [32,36]. As reported for most plant pathogens [37,38], it is difficult to explain how adaptations occurred in *E. turcicum*. However, it is known that the influence of weather conditions decreases when *E. turcicum* populations are more aggressive [39].

The DA selected the variables incubation period, disease severity at 19 dpi, sporulation in vivo, and spore production in vitro for classification of isolates according to their origin. DA only misclassified the Argentinian isolates A11-6, leading to the conclusion that the aggressiveness of this isolate is similar to that of European isolates. Climate data from the last 50 years show that Pergamino in Argentina (origin of A11-6) has temperate and very humid weather. Mean precipitation is above 1000 mm per year and the average temperature is 16 °C [40]. In Southeast Brazil, climate data from the last 30 years show average precipitation of around 1500 mm per year and an average temperature close to 21 °C [37]. The adaptation to mild temperatures of this Argentinian isolate may, therefore, explain why it was positioned with European isolates.

The increase in disease severity over time is probably due to lesion expansion [26,27], since controlled conditions were not favorable to sporulation, and consequently not favorable to secondary infections. Interestingly, in average sporulation in vivo was higher with South American isolates than for European isolates in all temperatures, while average spore production of South American isolates in vitro was higher only at 25 and 30 °C. The more vigorous sporulation of South American isolates provides evidence that they are more effectively propagating at higher temperatures than European isolates. Higher sporulation under high temperatures is usually not expected in nature, since higher temperatures are not favorable for spore survival and germination [25]. However, under high temperature the plant may be affected by heat stress and its defense thus weakened. Therefore, infection and host colonization, and consequently, sporulation might be favored under these conditions [41]. A further factor involved might be an increased phytotoxin production, such as monocerin [42] and HT-toxin [38] by the pathogen, which may suppress host resistance at higher temperatures [43,44]. A potential mechanism is dysfunction in the detoxification process under such conditions [45]. Unfortunately, the effect of high temperature on host resistance in the presence of the pathogen is difficult to analyze under experimental conditions and, therefore, heat stress was neglected in our experiments.

A shorter incubation period is usually correlated to higher aggressiveness. *Magnaporthe oryzae* isolates which showed a shorter incubation period had higher values in other aggressiveness components. Therefore, for this pathosystem, isolates which start epidemics early are more aggressive [46]. In the present study, a strong correlation was observed between AUMGC and spore production in vitro (data not shown). However, no correlation was established between the in vitro and in vivo variables. In the in vivo experiment, the factor host genotype was added. As distinct host genotypes have distinct resistance backgrounds, the response to the environment and pathogen isolate can be different [22]. However, maize lines and hybrids used in this study had similar levels of susceptibility. Thus, the host genotype effect was weak, as observed in the variance component analysis (VCA).

There were no similarities according to race or race complexity (data not shown). Race complexity reflects the number of different resistance genes that one isolate can overcome [47]. In nature, the emergence of complex races is unlikely to occur, unless there is a selection for more virulent populations by the cultivation of multi-resistant varieties. However, isolates bearing more virulence genes may not always be the most aggressive [24], and may or may not have fitness costs [47]. Nonetheless, it is not possible to make the same association for maize–*E. turcicum*, as aggressiveness is not correlated with pathogen fitness. A high sporulation rate does not imply a higher survival rate [24]. *Cochliobolus carbonum* and *C. heterostrophus* are pathogens that represent a trade-off between aggressiveness and fitness. Low aggressiveness levels and high survival ability were observed for *C. carbonum* which was the opposite of what has been observed for *C. heterostrophus* [24].

Overall, our study provides evidence for a strong impact of temperature regimes on vigor and aggressiveness of *E. turcicum* which in turn was related to the origin of isolates from a warmer or cooler climate. Isolates from warmer climates, corresponding to the optimal conditions for the host plant, when tested under equal conditions and on similar host genotypes, grew and sporulated more vigorously in vitro and were more aggressive on their host plant. This may indicate a longer and thus more successful adaptation to their host plants in warmer than in cooler conditions corresponding to the history of maize cultivation in tropical and moderate climates. Such adaptive shift to more aggressive fungal isolates may imply that maize cultivation in cooler climates will face more aggressive isolates under continued climate warming.

## 4. Materials and Methods

### 4.1. Exserohilum turcicum Isolates

Isolates selected for aggressiveness comparisons were chosen according to their provenance and race. Isolates were selected in order to have at least one isolate for each race complexity, from a single country. Isolates were obtained from race assessments conducted in Europe (*n* = 645) [10] and in South America (*n* = 184) [11]. Races were determined according to previous works [10,11]. Briefly, the race determination was conducted by inoculating a differential set of the maize line B37 without resistance genes (control) and B37 carrying the resistance genes *Ht1, Ht2, Ht3* and *Htn1*, as no molecular methods are established to determine the physiological race of *E. turcicum.* Maize plants were cultivated in a greenhouse (22 ± 6 °C, 70% air humidity, day/night light regime 14/10 h, light intensity 100 ± 20 µmol m^−2^ s^−1^). The race is determined based on the phenotype 14 days post inoculation. Plants displaying strong chlorosis are classified as resistant, whereas plants showing strong necrosis are susceptible [48]. Finally, 14 isolates (seven isolates from Europe and seven from South America) were selected for in vitro and in vivo tests. Race complexity is based on the number of *Ht* resistance genes which an isolate is able to overcome and cause disease (Table 1) [49].

### 4.2. In Vitro Tests

The in vitro experiments were performed for each isolate (Table 1) in order to observe the development of the pathogen under different temperatures. Mycelium growth and spore production were evaluated. All isolates, stored in fresh glycerin (25%) at −20 °C (up to 60 days), were transferred to Petri dishes containing V8 medium (75 mL V8 vegetable juice; 1.5 g CaCO_3_, 10 g agar-agar). After 28 days, mycelial plugs (3 mm-diameter) were transferred to V8 plates and grown under four temperatures (15 °C, 20 °C, 25 °C, or 30 °C) in the dark for 14 days. Each treatment was replicated four times (4 plates per isolate at each temperature). On each day after inoculation perpendicular measurements of the colony radius were taken from each plate. Finally, the AUMGC was calculated by trapezoidal integration adapted from Berger [50], according to the following formula:

$$\mathrm{AUMGC}=\overset{n-1}{\underset{1}{\sum }}\left(\frac{\;x_{i}+x_{i+1}}{2}\right)\left(t_{i+1}-t_{i}\right)$$

where $x_{i}\;$is the average colony radius for the measurement number i, $t_{i}\;$is the corresponding number of days of this observation, and *n* is the number of measurements. AUMGC was calculated from 1 to 7 days post inoculation (dpi), when the first plates were totally covered with mycelium. At 15 dpi, conidia were harvested from each plate, by washing with 10 mL of sterile distilled water and stored in Falcon tubes at −20 °C. Three aliquots from each spore suspension were counted using a hemocytometer; and the conidia production was calculated per plate. The in vitro experiments were repeated four times and four replicated plates were used.

### 4.3. In Vivo Tests

Maize plants from the near isogenic line (NIL) B37 (reference line used in race monitoring), Sus1 (highly susceptible line provided by breeders as a positive control), and the hybrid line Niklas^®^ (widely cultivated in Germany) were sown to test the aggressiveness of *E. turcicum* isolates (Table 1). Seeds were provided by KWS Saat SE (Einbeck, Germany). Two seeds per pot (11 × 11 × 10 cm^3^) were sown in a mixture of soil with proportions of 3:3:1 (clay: compost: sand). The plants were cultivated in a greenhouse at 24 ± 3 °C, 70% of air humidity, and a light/dark photoperiod of 14/10 h. Maize plants were inoculated about 30 days after sowing when the fifth and sixth leaves were unfolded. In order to prepare the conidia suspension, five plates of each isolate were inoculated and incubated at 25 °C in the dark for 21 days until conidia have developed. Conidia were collected using an aqueous solution containing 125 ppm of the surfactant Silwet Gold^®^ (Certis Europe B.V., Hamburg, Germany), and the suspension was adjusted to 1500 conidia mL^−1^ with a hemocytometer. Approximately 7 mL of conidia suspension were sprayed per plant that were maintained in a humidity chamber for 24 h. All plants were transferred to climate chambers (RUMED^®^ Rubbarth Apparate GmbH, Laatzen, Germany) under the following day/night temperature conditions: 30/25 °C, 25/20 °C, 20/15 °C, and 15/10 °C, with a light/dark photoperiod of 14/10 h, light intensity of 120 ± 10 µmol m^−2^ s^−1^ and relative air humidity of 70%. For each isolate, temperature and maize host, four replicated plants were inoculated. The in vivo experiments were repeated two times using four plants as technical replicates.

The comparisons of aggressiveness among isolates were based on the incubation period, disease severity, AUDPC and sporulation. The incubation period was evaluated when the plant showed the first lesion. Disease severity was evaluated every 3 days based on a diagrammatic scale ranging from 2 to 90% [51]. The final disease severity was obtained at 26 dpi. AUDPC was estimated by trapezoidal integration [50] according to the following formula:

$$\mathrm{AUDPC}=\overset{ni-1}{\underset{i=1}{\sum }}\left(\frac{\;y_{i}+y_{i+1}}{2}\right)\left(t_{i+1}-t_{i}\right)$$

where $y_{i}$ is the disease severity at the i evaluation, $t_{i}$ is time in days post inoculation at the *i*th evaluation, and *n* is the total number of evaluations.

Sporulation was measured on line B37 plants at 26 dpi. Symptomatic leaf samples of 6 cm^2^ (3 cm × 2 cm) from the fifth unfolded leaf were collected at the transition between green and necrotic areas. Samples of the four inoculated plants per treatment were placed on moistened filter paper (Munktell Ahlstrom) to maintain high humidity and stimulate conidia production. Pictures were taken of each sample to quantify the diseased area using Image J1.52a software (Wayne Rasband, National Institute of Health, Bethesda, MD, USA). The disease severity (%) of each sample was estimated using Assess 2.0 software (Lakhdar Lamari, 2008, APS, St. Paul, MN, USA). After 3 days, each sample was placed individually in a Falcon tube containing 4 mL of sterile distilled water amended with 125 ppm of the surfactant Silwet Gold^®^. Falcon tubes were frozen at −20 °C for further procedures. After mixing of the sample, sporulation was estimated using a haemocytometer. Three aliquots per sample were evaluated, sporulation was estimated from the average of these aliquots and divided by the diseased area, obtaining values of spores×cm^−2^.

### 4.4. Data Analysis

Data analysis of in vitro experiments was performed applying mixed models and estimations by the restricted maximum likelihood method using the lmer package of R 3.6.0 software (R Core Team, 2019). The in vitro experiments were completely randomized within the temperature treatments. Data of conidia production in vitro were analyzed with Box Cox transformation. Data were compared by ANOVA and multiple comparison applying Tukey test between isolates for each temperature (*p*-value ≤ 0.05).

In the in vivo experiments, a variance component analysis (VCA) was performed for the variables incubation period and AUDPC in order to assess the effect of the factors temperature, isolate origin and host genotype. Variance was estimated by the restricted maximum likelihood method and performed using the package VCA in R 3.6.0 software (R Core Team, 2019). As the factor temperature was showing the highest percentage of the total variance, an ANOVA was conducted per each temperature considering isolate origin as main effect and experiment replication and host genotypes as random effects. A second ANOVA was conducted per each temperature considering each single isolate as main effect and experiment replication and host genotypes as random effects. In addition, isolates were compared by multiple comparison applying the Tukey test (*p*-value ≤ 0.05). Data of incubation period and sporulation in vivo were analyzed after Box Cox transformation. As the host genotype was contributing to the variance, the effect of host genotype on AUDPC was compared between isolates origin and isolates for each temperature performing an ANOVA with experiment replication as random effect. Another ANOVA was conducted per each temperature considering each single isolate as main effect and experiment replication as random effects. In addition, isolates were compared by multiple comparison applying Tukey test (*p*-value ≤ 0.05).

Further analyzes were performed using data from the 30 °C experiment; data for the in vivo variables were chosen for the reference line B37 at 30/25 °C. The mean of each variable was calculated for each isolate. A stepwise DA was performed to identify which variables contributed most to differences between the two groups of isolates originating from Europe and South America. DA was performed with Statistica 13.0 software (Statsoft, Tulsa, OK, USA) by the forward method. Additionally, a PCA was performed with variables selected by the DA to show isolates position.

## 5. Conclusions

South American *E. turcicum* isolates grew more vigorously and were more aggressive than European isolates, since the values of most of the tested aggressiveness components (AUDPC, sporulation in vivo, AUMGC and spore production in vitro) were higher for South American isolates, especially at higher temperatures. The fact that *E. turcicum* isolates originating from regions with warmer temperatures are more aggressive than those from regions with milder temperatures implies a putative effect of longer co-evolution of pathogen and host under warmer conditions promoting adaptive shifts to more aggressiveness. Accordingly, temperature was the factor with the greatest influence on pathogen aggressiveness, since the tested temperature range was broad, from 15 to 30 °C. Although the host genotype is known to have a large effect on aggressiveness [24], in our study, the host genotype did not explain the variance because all three hosts were moderately susceptible to *E. turcicum*. The results from in vitro and in vivo experiments indicate that *E. turcicum* populations display considerable plasticity [32] and may adapt to the environmental conditions they are exposed to [21]. Adaptability to environmental conditions is an advantage for pathogen populations, in case of temperature increases due to climate warming or range expansion of the host crop. The latter has happened with maize in the last few decades when expanding to cooler climates in Europe where a warming climate may thus induce pathogen populations with increased aggressiveness.

## Figures and Tables

**Table 1:** *Exserohilum turcicum* isolates used for in vitro and in vivo tests.

Isolate	Continent	Country	Region	City	Race	Rc ^1^	Climate ^2^
D6-1	Europe	Germany	Bayern	Regensburg	Race 0	0	Dfb
HH138-1	Europe	France	Oberhein Region	Fessenheim	Race 1	1	Cfb
HH80	Europe	Belgium	East Flanders	Beervelde	Race 2	1	Cfb
D10-1	Europe	Germany	Niedersachsen	Meppen	Race 3	1	Dfb
D10-3	Europe	Germany	Niedersachsen	Meppen	Race 3N	2	Dfb
D3-3	Europe	Germany	Bayern	Regensburg	Race 123	3	Dfb
HH123-2	Europe	Turkey	Adana	Adana	Race 23N	3	Csa
B3-2	South America	Brazil	Paraná	Castro	Race 0	0	Cfb
A18-1	South America	Argentina	Entre Ríos	Victoria	Race 1	1	Cfa
B31-2	South America	Brazil	Rio Grande do Sul	Horizontina	Race 2	1	Cfa
A11-6	South America	Argentina	Missiones	Pergamino	Race 3	1	Cfa
B1-5	South America	Brazil	Paraná	Campo Largo	Race 12	2	Cfb
B10-1	South America	Brazil	Minas Gerais	Florestal	Race 23N	3	Cwa
B7-1	South America	Brazil	Minas Gerais	Sete Lagoas	Race 23N	3	Cwa

^1^ Race complexity (Rc) denotes the number of differential lines for which a specific isolate is virulent; rc1 = race complexity 1; rc2 = race complexity 2; rc3 = race complexity 3. ^2^ The climate from the region where each isolate was collected was classified as Cfa (temperate climate without dry season, with hot summer), Cfb (temperate climate without dry season, with warm summer), Cwa (temperate climate with dry winter and with hot summer), Dfb (cold climate without dry season with warm summer), or Csa (temperate climate with dry summer, with hot summer), according to the climate classification of Köppen-Geiger [8].

**Figure A1:**
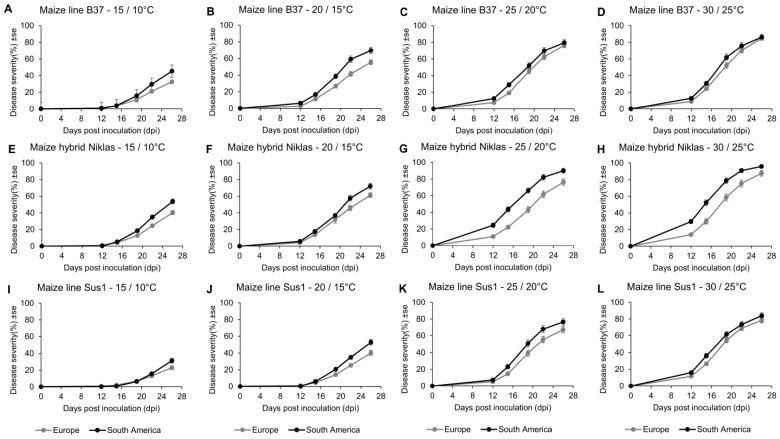
Disease severity of *Exserohilum turcicum* isolates from Europe and South America inoculated on three different maize hosts: B37–standard line for race monitoring (**A**–**D**), Niklas^®^–susceptible hybrid cultivated in Germany (**E**–**H**), and Sus1–susceptible maize line in breeding programs (**I**–**L**), and maintained under different day/night temperatures after inoculation: 30/25 °C (**A**,**E**,**I**), 25/20 °C (**B**,**F**,**J**), 20/15 °C (**C**,**G**,**K**), and 15/10 °C (**D**,**H**,**L**). Graphs represent the standard error of seven isolates in two experiments (*n* = 56).

## Data Availability

The raw data supporting the conclusions of this article will be made available by the authors, without undue reservation, to any qualified researcher.

## Additional Information

Brazilian isolates are in regulation to the Brazilian law n. 13,123, 20 May 2015 to access the genetic property, access number AB68A83, shipping number RFB2561.
